# Association of Neuropsychiatric Symptoms with Blood‐Based Biomarkers pTAU181, NFL, and GFAP in Alzheimer's Disease and Mild Cognitive Impairment

**DOI:** 10.1002/alz70856_103663

**Published:** 2025-12-25

**Authors:** Rahmah IKHLAS, Neda Rashidi‐Ranjbar, Nathan W. Churchill, Simon J. Graham, Tom A. Schweizer, Luis R Fornazzari, David G. Munoz, Corinne E. Fischer

**Affiliations:** ^1^ Keenan Research Centre for Biomedical Science, LKSI, St. Michaels Hospital, Toronto, Canada, Toronto, ON, Canada; ^2^ Keenan Research Centre for Biomedical Science, Li Ka Shing Knowledge Institute, St. Michael's Hospital, Toronto, ON, Canada; ^3^ Sunnybrook Research Institute, Toronto, ON, Canada; ^4^ Department of Medical Biophysics, University of Toronto, Toronto, ON, Canada; ^5^ Division of Neurosurgery, St. Michael's Hospital, Toronto, ON, Canada; ^6^ Institute of Medical Science, Temerty Faculty of Medicine, University of Toronto, Toronto, ON, Canada; ^7^ Division of Neurosurgery, Faculty of Medicine, University of Toronto, Toronto, ON, Canada; ^8^ Department of Medicine, Neurology Division, University of Toronto, Toronto, ON, Canada; ^9^ Temerty Faculty of Medicine, Department of Laboratory Medicine and Pathobiology, University of Toronto, Toronto, ON, Canada; ^10^ Division of Pathology, St. Michael's Hospital, Toronto, ON, Canada; ^11^ Temerty Faculty of Medicine, Department of Psychiatry, University of Toronto, Toronto, ON, Canada

## Abstract

**Background:**

Neuropsychiatric Symptoms (NPS) in Alzheimer's Disease (AD) can exacerbate symptom burden and impair quality of life in patients. It is imperative to gain a better understanding of factors that contribute to NPS manifestation. This study explores the association between NPS scores and blood‐based biomarkers implicated in AD including Glial Fibrillary Acidic Protein (GFAP), Neurofilament Light Chain (NFL), Amyloid Beta 40 (AB40), Amyloid Beta 42 (AB42), and Phosphorylated Tau at Threonine 181 (pTAU181).

**Methods:**

Individuals with a diagnosis of Alzheimer's Disease (AD) or Mild Cognitive Impairment (MCI) were included based on data from the Ontario Neurodegenerative Disease Research Initiative (ONDRI) database. From 126 participants, 107 had completed the Neuropsychiatric Inventory Questionnaire (NPI‐Q) and provided blood samples for measurement of GFAP, NFL, AB40, AB42, and pTAU181 plasma levels. To assess the association between NPS severity scores and blood‐based biomarkers, a partial Spearman correlation was conducted; adjusting for age, sex, and education level. A False Discovery Rate (FDR) threshold of 0.05 was applied to all *p*‐values to control for multiple comparisons.

**Results:**

We found significant correlations between several NPS domains and blood‐based biomarkers. *Apathy* scores had a significant positive correlation with pTAU181 (*Rs* = 0.27, *p* =  0.04) and NFL plasma levels (*Rs* =0.30, *p* =  0.02); Anxiety scores showed a significant positive correlation with GFAP (*Rs* = 0.28, *p* =  0.03) and NFL plasma levels (*Rs* = 0.33, *p* =  0.006); Euphoria scores were positively correlated with NFL plasma levels (*Rs* = 0.27, *p* =  0.05); Appetite, Motor function, and *Total NPI severity scores* had a significant positive correlation with GFAP plasma levels (*Rs* = 0.31, *p* =  0.01; Rs = 0.31, *p* =  0.01; Rs = 0.29, *p* =  0.03). No significant findings were observed for AB40 and 42 with any NPS domain.

**Conclusion:**

Our findings suggest that certain blood‐based biomarkers—pTAU181, NFL, and GFAP— are associated with the development of NPS in Alzheimer's Disease. These biomarkers may help shed light on underlying mechanisms and monitor NPS severity. However, further research in larger cohorts and longitudinal studies is needed to confirm these findings and explore causal relationships.

